# Supplementary material for: METTL14 regulates proliferation and differentiation of duck myoblasts through targeting MiR-133b
Source: PLoS One. 2025 Mar 28;20(3):e0320659. doi: 10.1371/journal.pone.0320659 (PMC11952261; doi:10.1371/journal.pone.0320659)
Supplement: File S1 — (DOCX) [file pone.0320659.s001.docx]

Table S1 Primer sequence

| Primer Name | Primer Sequence（5’→3’） | Fragment Size (bp) |
| --- | --- | --- |
| CDK2-F | F: AGGTGGTGACGCTGTGGTA | 169 |
| CDK2-R | R: GTGCGGAAGATGCGGAAGA |  |
| CyclinD1-F | F: TTCCTCTCCTATCAATGCCTCACA | 171 |
| CyclinD1C-R | R: GTCTGCTTCGTCCTCTACAGTCTT |  |
| MyHC-F | F: AAGAAGGCCATCACTGACGC | 527 |
| MyHC-R | R: ATTTTGGTGTGAACCTCGCG |  |
| MYOG-F | F: CCCACAATCTGCACTCCCTT | 572 |
| MYOG-R | R: CAGAGGCTTTGGAACCGGAT |  |
| METTL14-F | F: TGTCTACGCAAATGGGGTTA | 169 |
| METTL14-R | R: GGCAGTGCTCCTTGGTTCT |  |
| β-actin-F | F: GCTATGTCGCCCTGGATTTC | 127 |
| β-actin-R | R: CACAGGACTCCATACCCAAGAA |  |

Table S2 Primer sequence of miRNAs

| MiRNA Primer Name | Primer Sequence（5’→3’） |
| --- | --- |
| bta-miR-133b_R-1 | TTTGGTCCCCTTCAACCAGCT |
| bta-miR-26a R+3 | GCTTCAAGTAATCCAGGATAGGCT |
| mmu-miR-135a-5p | GCGGCCTATGGCTTTTTATTCCT |
| eca-miR-206 | GCTGGAATGTAAGGAAGTGTGTGG |
| has-miR-133a-5p | GCAGCTGGTAAAATGGAACCAAAT |
| has-miR-148a-3p_R-2 | GCCTCAGTGCACTACAGAACTTT |
| U6 | GGAACGATACAGAGAAGATTAGC |
| Universal downstream | R: TGGAACGCTTCACGAATTTGCG |

Table S3 Primer sequence

| Primer Name | Primer Sequence(5’→3’) | Fragment Size(bp) |
| --- | --- | --- |
| MET1S | GGCGTGGAGATGAACAG | 1400 |
| MET1AS | CCTAATAGTCATCAGCGTG |  |
| MET2S | GGCGGAATTCAGATGAACAGCCGCCT | 1389 |
| MET2AS | ATAGGGATCCGCGTGTGGGGAAGCCT |  |
| EGFNS | CGCAAATGGGCGGTAGGCGTG | 1585 |
| EGFNAS | CGTCGCCGTCCAGCTCGACCAG |  |

| Primer Name | Primer Sequence(5’→3’) | Fragment Size(bp) |
| --- | --- | --- |
| sh-METTL14-F | GATCCGGTCTAAGTTTCCCGAATTATTTCAAGAGAATAATTCGGGAAACTTAGACCTTTTTTG | 63 |
| sh-METTL14-R | AATTCAAAAAAGGTCTAAGTTTCCCGAATTATTCTCTTGAAATAATTCGGGAAACTTAGACCG |  |
| NC-F | GATCCGTCAGTACATTGCATCGACAGTTCAAGAGACTGTCGATGCAATGTACTGACTTTTTTG | 63 |
| NC-R | AATTCAAAAAAGTCAGTACATTGCATCGACAGTCTCTTGAACTGTCGATGCAATGTACTGACG |  |

Table S4 METTL14 interference sequence
