# Supplementary material for: METTL14 regulates proliferation and differentiation of duck myoblasts through targeting MiR-133b
Source: PLoS One. 2025 Mar 28;20(3):e0320659. doi: 10.1371/journal.pone.0320659 (PMC11952261; doi:10.1371/journal.pone.0320659)
Supplement: File S2 — (DOCX) [file pone.0320659.s002.docx]

**Table S5** miRNAs related to skeletal muscle development

| miR_name | miR_seq |
| --- | --- |
| hsa-miR-133a-5p | AGCTGGTAAAATGGAACCAAAT |
| mmu-miR-135a-5p | TATGGCTTTTTATTCCTATGTGA |
| hsa-miR-148a-3p_R-2 | TCAGTGCACTACAGAACTTT |
| eca-miR-206 | TGGAATGTAAGGAAGTGTGTGG |
| mdo-miR-26-5p_R+1 | TTCAAGTAATCCAGGATAGGCT |
| bta-miR-133b_R-1 | TTTGGTCCCCTTCAACCAGCT |
